# Value of Infrared Thermography Camera Attached to a Smartphone for Evaluation and Follow-up of Patients with Graves' Ophthalmopathy

**DOI:** 10.1155/2019/7065713

**Published:** 2019-05-09

**Authors:** Cínthia Minatel Riguetto, Walter José Minicucci, Arnaldo Moura Neto, Marcos Antonio Tambascia, Denise Engelbrecht Zantut-Wittmann

**Affiliations:** Endocrinology Division, Faculty of Medical Sciences, University of Campinas, Rua Tessália Vieira de Camargo 126, 13084-971 Campinas, São Paulo, Brazil

## Abstract

**Purpose:**

Graves' ophthalmopathy (GO) is the most common extra-thyroid manifestation of Graves' disease (GD). The Clinical Activity Score (CAS) has been widely used to evaluate GO inflammation severity and response to treatment; however, it is quite subjective. Infrared thermography (IRT) is a portable and low-cost device to evaluate local temperature and assess inflammation. The aim was to evaluate ocular temperature by IRT as an instrument for measuring inflammatory activity in GO and its correlation with CAS.

**Methods:**

This is a cross-sectional study involving 136 consecutive GD patients (12 with CAS ≥ 3/7, 62 with CAS < 3 and 62 without apparent GO) with 62 healthy controls. Patients with active ophthalmopathy were prospectively evaluated. Exophthalmometry, CAS, and thermal images from caruncles and upper eyelids were acquired from all subjects.

**Results:**

All eye areas of thermal evaluation had higher temperatures in GD patients with active ophthalmopathy (caruncles,* p<0.0001*; upper eyelids,* p<0.0001*), and it was positively correlated with CAS (r=0.60 and* p<0.0001 a*t caruncles; r=0.58 and* p<0.0001* at upper eyelids). No difference in temperature was found between other groups. Patients with active ophthalmopathy were prospectively evaluated after 6 or 12 months of the treatment and a significant difference was found in ophthalmometry (*p=0.0188*), CAS (*p=0.0205*), temperature of caruncles (*p=0.0120*), and upper eyelids (*p=0.0066*).

**Conclusions:**

IRT was an objective and simple tool for evaluation and follow-up of inflammation in GO, allowed evidencing patients with significant inflammatory activity, and had a good correlation with the CAS score.

## 1. Introduction

Graves' ophthalmopathy (GO) is the most common and serious extra-thyroid manifestation of Graves' disease (GD), affecting about 50% of patients, and is usually bilateral, but may be unilateral [1, 2]. The signs and symptoms related to GO are eyelid retraction, ocular irritation, photophobia, dry eye, increased tearing, conjunctival redness, eyelid swelling, diplopia, ocular pain, ptosis, periorbital edema, proptosis, and even sight loss [3].

The assessment of GO is performed through physical examination, to determine the ocular inflammation degree and proptosis [4]. Proptosis can be evaluated by exophthalmometry, with a simple tool to measure the distance between the outer corner of the eye and the cornea. Disease activity can be graded by Clinical Activity Score (CAS), introduced in 1989 by Mourits et al. [5, 6], that was developed to predict which patient would have a better response to immunosuppressive treatment. The scale evaluates soft tissue inflammation and assigns 1 point to each of the following manifestations: spontaneous orbital pain, gaze-evoked orbital pain, eyelid swelling, eyelid erythema, conjunctival redness, chemosis, and inflammation of caruncle or plica. A CAS of 3 or higher indicates active disease. For patient's follow-up, 3 more items are included in CAS evaluation, which is increase of ≥ 2 mm in proptosis, decrease in the ocular excursion in any one direction of ≥ 8° and a decrease of acuity equivalent to 1 Snellen line. Although CAS has been widely used, it is partly subjective, depending on the experience of each physician and either mild or severe ophthalmopathy is calculated with the same point.

Several imaging techniques are currently used to access GO, such as ultrasound, computer tomography, and magnetic resonance, though these techniques are expensive, are poorly available, depend on experienced technician, and can expose patients to contrast agents and radiation [7–9]. In the search for a more accessible and objective method to evaluate disease activity in GO, Infrared Thermography (IRT) appears to be a promising tool and is gaining importance in medical community due to recent studies and different applications for this new device [10]. It has been used in several conditions, such as breast cancer, monitoring of fracture consolidation, the evolution of pressure ulcers, diabetic foot, and burns [11–15]. In GO, Chang et al. [16] and Shih et al. [17] were pioneers to evaluate IRT during follow-up of patients treated with methylprednisolone pulse. What makes IRT even more interesting is the fact that the device used is simple and portable, has a relatively low cost (approximately $300 or €250), and can be coupled to a smartphone, allowing a comparison between temperatures during follow-up.

The primary aim of the study was to evaluate ocular temperature by IRT as an instrument for measuring inflammatory activity in GO and its correlation with CAS.

## 2. Materials and Methods

### 2.1. Patients Recruitment

This is a cross-sectional clinic-based study involving 136 consecutive GD patients followed at the Thyroid Disease Unit at a tertiary Service of Endocrinology and a control group composed of 62 healthy individuals. One hundred and ninety-eight subjects were included (58 men and 140 women, median age 48.5 ± SD 14.39 years) and divided into 4 groups according to clinical characteristics. Basically, 12 patients present with GD with active ophthalmopathy (CAS ≥ 3/7), 62 patients with GD and inactive ophthalmopathy (CAS < 3), 62 patients with GD and without apparent ophthalmopathy, and 62 healthy controls with normal thyroid function and from an iodine sufficient area. Patients from the group of active ophthalmopathy were reassessed during follow-up. Exclusion criteria were any acute infectious or inflammatory disease, history of recent cardiovascular events (myocardial ischemia, unstable angina, and stroke), malignant neoplasia, heart failure (NYHA III or IV), severe hepatic disease, severe kidney disease (Chronic Kidney Disease stages 4 and 5 and hemodialysis), hepatitis B, C, and HIV infection, and any other acute or chronic ocular diseases [18].

Data were collected from August 2017 to March 2018, and all participants gave their written informed consent and ethical committee approval for the study was obtained according to Declaration of Helsinki, Human Research Ethics Committee in Lausanne, No. 204/14). Written informed consent for publication of their clinical details and/or clinical images was obtained from the patient (CAAE: 71204117.2.0000.5404).

### 2.2. Clinical Assessment

Clinical characteristics and biochemical data were recorded by chart review. Clinical data collected were age, age at diagnosis, gender, smoking habits, use of levothyroxine or methimazole, treatment with radioiodine, comorbidities, including other autoimmune or chronic disease, disease duration, and time of follow-up. Biochemical data collected were serum thyroid stimulating hormone (TSH) (reference values RV 0,41–4,5 mUI/L), free thyroxine (fT4) (RV 0,9–1,8 m/dL), thyroglobulin antibodies (TgAb) (RV < 115 mUI/L), thyroid peroxidase antibodies (TPOAb) (RV < 35 UI/mL), and TSH receptor antibodies (TRAb) (RV < 1,58 UI/mL) were measured by electrochemiluminescence immunoassay [18].

Clinical eye evaluation was elaborated to define the degree of ophthalmopathy activity, the measure of proptosis, and temperature by IRT. CAS assessed the degree of inflammation and was calculated from 7 items, with 1 point assigned to each alteration presented: spontaneous orbital pain, gaze-evoked orbital pain, eyelid swelling, eyelid erythema, conjunctival redness, chemosis, and inflammation of caruncle or plica. A CAS of 3 or higher indicates an active ophthalmopathy. For patients' follow-up, 3 more items are included in CAS evaluation, which is increase of ≥ 2 mm in proptosis, decrease in the ocular excursion in any one direction of ≥ 8°, and a decrease of acuity equivalent to 1 Snellen line. We did not use the additional points on follow-up visits of patients with active ophthalmopathy [18].

Proptosis was evaluated with an exophthalmometer routinely used in our service. It is an instrument composed of a lateral rod with marking in centimeters as a ruler that connects to a front rod, forming an angle of 90°. The lateral rod is adjusted to the temporal region of the patient, and then it is possible to measure the distance between the outer corner of the eye and the cornea [18].

Local temperature of the caruncle (right and left) and upper eyelid (right and left) were obtained using a FLIR ONE camera (FLIR Systems, Inc., Wilsonville, OR, USA) attached to an iPhone 6 (Apple, Inc., Cupertino, CA, USA). Images were analyzed in the FLIR Tools application. All subjects were advised not to smoke, drink alcohol or coffee, or use make-up 24 hours before the exam. They were evaluated in a calm, quiet, and controlled temperature room (set at 24°C), with no interference from the outside. They were comfortably seated on a stretcher, and after 10 minutes of rest, clinical evaluation with CAS and exophthalmometry was done. After that, the thermometer sensor was focused on the interest areas, at a distance of 20 centimeters, and images with temperature were captured. Firstly, the right eye (caruncle and upper eyelid) and then the left eye (caruncle and upper eyelid) were evaluated. Patients were advised not to blink and move during the examination. The three evaluations were done on the same day, one followed by the other, by the same physician.

Patients with active ophthalmopathy were prospectively reassessed with CAS, ophthalmometry, and IRT at caruncles and upper eyelids after at least 6 months of treatment, by the same physician and technique described above.

### 2.3. Statistical Analysis

Statistical analyses were carried out in the Statistical Analysis System (SAS), System for Windows, version 9.4. SAS Institute Inc., 2002-2008, Cary, NC, USA. To describe sample profile according to the study variables, frequency tables of categorical variables with absolute (n) and percentage (%) values were used, and descriptive statistics of numerical variables, with median (interquartile range). To compare categorical variables, the Qui-square test and, when necessary, Fisher's exact test were used. Mann-Whitney* U* test and Kruskal-Wallis test were used to compare numerical variables, followed by the Dunn post hoc test to identify the differences. To correlate the numerical variables with temperatures, Spearman correlation coefficient was used. To compare variables between the two evaluations of patients with active GO, ANOVA was used for repeated measures. Data were transformed into ranks. The significance level was set at p < 0.05.

## 3. Results

We analyzed 198 patients, of which 12 (6.06%) had GD with active ophthalmopathy (CAS ≥ 3/7), 62 (31.31%) had GD with inactive ophthalmopathy (CAS < 3), 62 (31.31%) had GD without ophthalmopathy, and 62 (31.31%) healthy controls.  Table 1 summarizes demographic and clinical variables from each group. There were no significant differences of gender, age at diagnosis, age at the evaluation, thyroid disease duration (time between diagnosis and current evaluation), time of follow-up, presence of TgAb and TPOAb, comorbidities (chronic or other autoimmune diseases), and radioiodine treatment between groups.

The group with active ophthalmopathy had a higher rate of a detectable TRAb (median 91.67% versus 79.1% and 78.43%,* p<0.0001*) and methimazole users (83.33% versus 30.65 and 40.32%,* p=0.0029*) than the inactive ophthalmopathy and without ophthalmopathy groups of GD, respectively. This group also have a lower TSH value (median 0.09 versus 1.87, 1.65, and 2.16,* p=0.0281*), a higher percentage of patients with history of smoking (41.67% versus 37.10%, 25.81%, and 11.29%,* p=0.0057*), as well as in currently smoking (33.33% versus 20.97%, 12.90%, and 4.84%,* p=0.0146*) than the patients with inactive ophthalmopathy, patients without ophthalmopathy and healthy control group, respectively.

GD patients with inactive ophthalmopathy had a higher percentage of levothyroxine users (58.06% versus 8.33% and 46.77%,* p=0.0064*) than the active ophthalmopathy and without ophthalmopathy groups, respectively. The healthy control group had a lower fT4 value when compared with the GD patient groups (median 0.99 versus 1.4, 1.32 and 1.29,* p<0.0001*).

The group of GD patients with ophthalmopathy, active or inactive, had significantly greater measures of proptosis when compared to GD patients without ophthalmopathy and healthy controls (20.25 and 13.5 mm versus 9.5 and 9.5 mm,* p<0.0001*).

**Figure 1:**
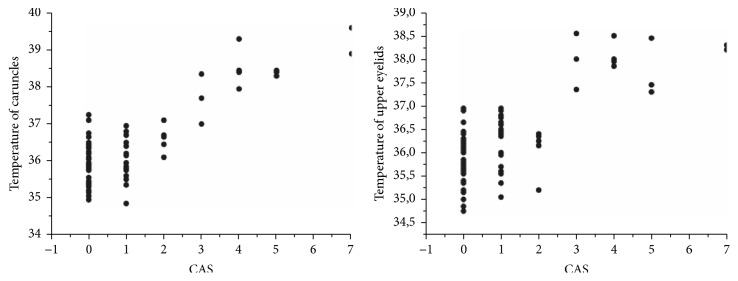
*Spearman's correlation graph between Clinical Activity Score (CAS) and temperature at caruncles and upper eyelids*. Caruncles: r=0.60,* p<0.0001 and* statistical power of 81.6%; Upper eyelids: r=0.58,* p<0.0001* and statistical power of 80.5%.

All eye areas of thermal evaluation had higher temperatures in GD patients with active ophthalmopathy when compared to other groups (caruncles 38.4 versus 36.05, 36.13, and 36.13°C,* p<0.0001*; upper eyelids 38 versus 36.08, 36.28, and 36.05°C,* p<0.0001*); moreover, it was positively correlated with CAS score in Spearman's correlation (r=0.60,* p<0.0001 and* statistical power of 81.6% at caruncles; r=0.58,* p<0.0001* and statistical power of 80.5% at upper eyelids), shown in Figure 1. No significant difference in temperature was found between GD patients with inactive ophthalmopathy, GD patients without apparent eye disease, and healthy control. In addition to the basic analysis between groups, patients with inactive ophthalmopathy were subdivided into CAS 0 and CAS 1-2 to verify if there was any temperature difference between those who had some degree of orbital inflammation and those with no sign of activity; however, no significant difference was found.  Table 2 summarizes the comparative analysis of ophthalmometry, CAS score and temperatures at caruncles and upper eyelids between groups.

**Figure 2:**
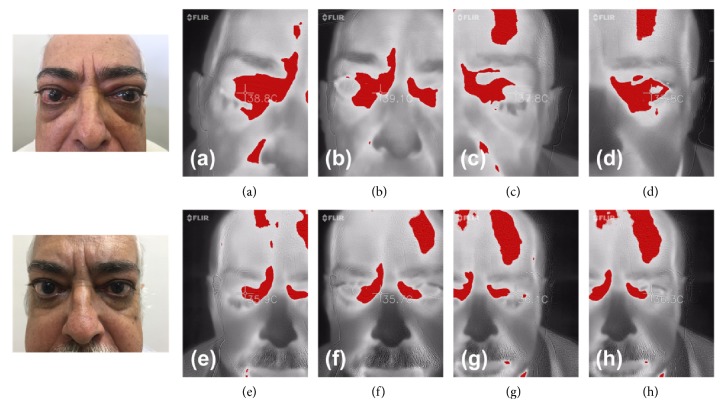
*Prospective evaluation of infrared thermography of a patient with active Graves' Ophthalmopathy*. Example of thermal images from a male patient, 60-year-old, with GD and active ophthalmopathy, currently using 20 mg of methimazole. At first physical exam: CAS of 7, measure of right eye and left eye was 17 and 15 mm, respectively. TSH and fT4 were, respectively, 0.07 mUI/L and 2.33 m/dL. Thermal evaluation: (a) 38.8°C, right upper eyelid; (b) 39.1°C, right caruncle; (c) 37.8°C, left upper eyelid; (d) 37.8°C, left caruncle. The patient was treated with prednisone 40 mg daily and local care with eyedrops. Methimazole dose was increased and after 6 months orbital decompression surgery was performed in both eyes. At second physical exam: CAS of 2, measure of right eye and left eye was 15 and 12 mm, respectively. TSH and fT4 were, respectively, 1.46 mUI/L and 1.44 m/dL. Thermal evaluation: (e) 35.9°C, right upper eyelid; (f) 35.7°C, right caruncle; (g) 36.1°C, left upper eyelid; (h) 36.3°C, left caruncle.

All patients with active ophthalmopathy were prospectively evaluated after at least 6 months of treatment employed in each case, and a significant difference was found in exophthalmometry (median before treatment 20.25 mm and after treatment 13.75 mm,* p=0.0188*), CAS (median before treatment 4 and after treatment 1.5,* p=0.0205*), temperature of caruncles (median before treatment 38.4°C and after treatment 36.58°C,* p=0.0120*), and temperature of upper eyelids (median before treatment 38°C and after treatment 36.48°C,* p=0.0066*). No significant difference was found in TSH and fT4 at prospective analysis.  Table 3 shows the statistics differences before and after treatment described above and Table 4 summarizes clinical characteristics, time of follow-up, and treatment employed in all patients with active ophthalmopathy.  Figure 2 shows the prospective thermal evaluation of a male patient, before and after the treatment with prednisone and orbital decompression surgery.

## 4. Discussion

GO results of the enlargement of extraocular muscles and retrobulbar fat lead to an increase in intraorbital pressure [19, 20]. As a consequence, proptosis and the reduction of venous drainage result in periorbital edema, conjunctival edema, and conjunctival hyperemia. For proper clinical management, it is essential that activity and severity of GO be determined to choose the better therapy for each patient [21, 22]. In milder cases, with the presence of minimal inflammation, local cares may be sufficient. However, for active GO with moderate to severe inflammation, anti-inflammatory and immunomodulatory treatment are necessary [23, 24].

Establishing the degree of inflammation and treating it with anti-inflammatory and immunomodulatory drugs are the basis of GO treatment [25], though the tools available are subjective and cannot express the severity of each sign and symptom. The CAS score is useful and widely used in clinical practice including our tertiary hospital; nevertheless the evaluation of their items is relatively subjective since it gives the same value to each sign and symptom presented, regardless of its severity and did not distinguish the score separately in each eye. Another important point is the fact that the original CAS score do not evaluate heat as part of the inflammatory signs, probably due to the difficulty to measure temperature when the score was created. Nowadays, we have precise devices to add this valuable information in clinical examination and follow-up.

In seeking to find a more objective and assessment tools, IRT seems to be an interesting technique to evaluate patients with GO, since inflammation is the basis of eye disease physiopathology and heat is associated with it. Chang et al. [16] and Shih et al. [17] have shown that IRT, combining with CAS, could better predict outcome of the use of methylprednisolone in active GO and is also useful to patients follow-up, evidencing a decrease in ocular temperature after improvement of eye inflammation. Another most recent study from Di Maria et al. [26] compared five novel thermal eye parameters in 17 patients with active thyroid eye disease (CAS > 3/7) and 13 with inactive disease (CAS < 3). They also found higher temperatures in patients with active eye disease.

In this study, we compared clinical characteristics with CAS score and temperatures of caruncles and upper eyelids in 3 distinct populations of patients with GD, according to the activity of thyroid disease and ophthalmopathy, in addition to the healthy control group [27]. The decision to analyze patients with and without ophthalmopathy was based on studies that showed eye alterations on orbital magnetic resonance images, even in patients without apparent ophthalmopathy [28].

We found higher temperatures among patients with active ophthalmopathy and it was positively correlated with CAS score with significant statistic power. Although orbital alterations were reported in imaging studies of patients without apparent ophthalmopathy, no difference in temperature was found when we compared the groups with inactive ophthalmopathy, without ophthalmopathy and healthy controls, probably because they had a discrete or no sign of orbital inflammation. We also found no difference among patients with inactive ophthalmopathy and CAS of 1 or 2, which shows that temperature assessment may be more useful for patients with active ophthalmopathy since it correlates very well with more evident signs of inflammation. As an example, we can observe that patients with active ophthalmopathy had a minimum temperature of 37°C and a median of 38°C, while patients with inactive ophthalmopathy, without ophthalmopathy and controls, had a minimum of 34. This fact shows that the thermal evaluation per se could indicate an orbital inflammatory activity [27].

Patients who were prospectively analyzed presented a decrease in proptosis, CAS and ocular temperature after the treatment used for each case, associated with improvement of signs of orbital inflammation and symptoms. Despite the evidence of clinical improvement of patients with active ophthalmopathy, the use of IRT was a great help in patients follow-up, since it is a portable and friendly device, can record images during the various evaluation, and can correlate symptoms and CAS with orbital temperature [27]. The idea is not to replace the clinical score for a device but to have a more objective tool in association with the classic evaluation already used and thus to determine the better follow-up for each patient.

As for the pitfalls of this study, first of all, there is a small sample of patients, especially the group with active ophthalmopathy and the analysis of just 2 thermal areas in each eye (caruncle and upper eyelid), instead of 5 or 6 thermal areas in other studies. However, the 2 analyzed thermal areas found a similar result than the analysis in multiple areas, being more practical and fast to carry out. Another point is that we did not evaluate body temperature because all of our samples were obtained of patients from outpatient clinics and have no evidence of other acute diseases than GO. In contrast to the pitfalls, our study is the first to analyze 3 distinct populations of patients with GD and a healthy control group.

In conclusion, we found that IRT in association with CAS was an excellent evaluation mechanism for patients with active eye disease. Additionally, IRT device is an objective, simple, and portable tool that can be coupled to a smartphone, allowing a comparison between temperatures and images during follow-up.

## 5. Conclusion

Our study demonstrated that IRT was an objective and simple tool for evaluation and follow-up of inflammation in GO and had a good correlation with severity of CAS score. However, a large-scale investigation is indispensable to confirm our results.

## Figures and Tables

**Table 1:** Baseline characteristics of 136 patients with Graves' disease and 62 healthy controls.

	GD patients with active ophthalmopathy (CAS > 3)	GD patients with inactive ophthalmopathy (CAS < 3)	GD patients without ophthalmopathy	Healthy controls	*p Value*
	N = 12	N = 62	N = 62	N = 62	
Gender (female/male)	5 / 7	45 / 17	45 / 17	45 / 17	0.1576
Age at the evaluation (years)	53 (15 – 68)	48 (19 – 80)	49.5 (16 – 80)	48 (15 – 80)	0.9937
Age at diagnosis (years)	45.5 (10 – 66)	40 (10 – 63)	41.5 (11 – 78)		0.6836
Thyroid disease duration (years)	2.5 (1 – 8)	6.5 (1 – 42)	4.0 (1 – 27)		0.0822
Follow-up (years)	2 (1 – 7)	5 (1 – 23)	4 (1 – 25)		0.2703
TSH at the evaluation (mUI/L)	0.09 (0.01 – 6.05)	1.87 (0.01 – 6.75)	1.65 (0.01 – 6.02)	2.43 (0.49 – 5.64)	*0.0281*
fT4 at the evaluation (m/dL)	1.4 (0.65 – 3.59)	1.32 (0.77 – 4.88)	1.29 (0.56 – 3.21)	0.99 (0.61 – 1.48)	*<0.0001*
TgAb (> 115 mUI/L)	2 (20%)	19 (33.33%)	27 (46.55%)		0.1589
TPOAb (> 35 UI/mL)	4 (40%)	38 (63.33%)	43 (74.14%)		0.0848
TRAb (> 1,58 UI/mL)	11 (91.67%)	38 (79.17%)	40 (78.43%)		*<0.0001*
Comorbidities					0.7200
Chronic diseases*∗*	5 (41.67%)	25 (40.32%)	28 (45.16%)	33 (53.23%)	
Other autoimmune diseases*∗∗*	0	2 (3.23%)	4 (6.45%)	3 (4.84%)	
History of smoking	5 (41.67%)	23 (37.10%)	16 (25.81%)	7 (11.29%)	*0.0057*
Currently smoking	4 (33.33%)	13 (20.97%)	8 (12.90%)	3 (4.84%)	*0.0146*
Radioiodine treatment	2 (16.67%)	21 (33.87%)	26 (41.94%)		0.2214
Patients using methimazole	10 (83.33%)	19 (30.65%)	25 (40.32%)		*0.0029*
Patients using levothyroxine	1 (8.33%)	36 (58.06%)	29 (46.77%)		*0.0064*

GD, Graves ‘disease; CAS, Clinical Activity Score; N, number; TSH, thyroid stimulating hormone; fT4, free thyroxine; TgAb, thyroglobulin antibodies; TPOAb, thyroid peroxidase antibodies.

Values are reported as median (lower quartile – upper quartile) or counts. The P value indicates if any statistically significant difference was found between groups. Statistically significant P values are in italic.

*∗*Hypertension, diabetes, dyslipidemia, and obesity. *∗∗*Vitiligo, celiac disease, and rheumatoid arthritis.

**Table 2:** Comparative analysis of ophthalmometry, CAS, and temperatures of caruncles and upper eyelids between groups.

	GD patients with active ophthalmopathy (CAS > 3)	GD patients with inactive ophthalmopathy (CAS < 3)	GD patients without ophthalmopathy	Healthy controls	*p Value*
Ophthalmometry (mm)	20.25 (13 – 27.5)	13.5 (7 – 22.5)	9.5 (7 – 13)	9.5 (7 – 12,5)	*< 0.0001*
CAS	4 (3 – 7)	0 (0 – 2)			
Temperature of caruncles (°C)	38.4 (37 – 39.6)	36.05 (34.85 – 37.25)	36.13 (34.3 – 37.4)	36.13 (34.35 – 37.35)	*< 0.0001*
Temperature of eyelids (°C)	38 (37.3 – 38.55)	36.08 (34.75 – 36.95)	36.28 (33.3 – 37.15)	36.05 (34.35 – 37.2)	*< 0.0001*

GD, Graves ‘disease; CAS, Clinical Activity Score; mm, millimeters; °C, celsius.

Values are reported as median (lower quartile – upper quartile) or counts. The P value indicates if any statistically significant difference was found between groups. Statistically significant P values are in italic.

**Table 3:** Clinical and laboratory characteristics of the prospective analysis of the 12 patients with active ophthalmopathy.

	First evaluation	Second evaluation	*p Value*
	N = 12	N = 12	
TSH (mUI/L)	0.09 (0.01 – 6.05)	1.68 (0.01 – 4.29)	0.4742
fT4 (m/dL)	1.40 (0.65 – 3.59)	1.12 (0.70 – 2.05)	0.6652
Ophthalmometry (mm)	20.25 (13 – 27.5)	13.75 (11 – 27.5)	*0.0188*
CAS	4 (3 – 7)	1.5 (0 – 5)	*0.0205*
Temperature of caruncles (°C)	38.4 (37 – 39.6)	36.58 (35.55 – 37.9)	*0.0120*
Temperature of upper eyelids (°C)	38 (37.3 – 38.55)	36.48 (35.7 – 37.6)	*0.0066*

GD, Graves ‘disease; N, number; TSH, thyroid stimulating hormone; fT4, free thyroxine; mm, millimeters; CAS, Clinical Activity Score. Values are reported as median (lower quartile – upper quartile) or counts. The P value indicates if any statistically significant difference was found between the two evaluations. Statistically significant P values are in italic.

**Table 4:** Prospective clinical and thermographic evaluation of the 12 patients with Graves' disease and active ophthalmopathy.

		Clinical Activity Score before and after treatment	Ophthalmometry before and after treatment (mm - millimeters)	Temperature of right upper eyelid, right caruncle, left upper eye lid and left caruncle before and after treatment (°C – degree Celsius)	Time between the first and second evaluation	Treatment employed
Patient 1: 15-year-old male	Before After	4/7 0/7	right - 20/left - 18 right - 11/left - 14	38.7; 39.3; 37.3; 37.6 36.7; 36.2; 36.5; 36.6	6 months	Methimazole and eyedrops

Patient 2: 15-year-old female	Before After	3/7 0/7	right - 25/left - 25 right – 24/left - 22	38.5; 38; 37.5; 37.4 36.1; 35.3; 36.6; 36.4	12 months	Methimazole and eyedrops

Patient 3: 61-year-old male	Before After	4/7 3/7	right - 20/left - 18 right - 30/left - 25	39.3; 39.1; 38.3; 38.1 37.8; 37.7; 37.3; 37.5	12 months	Methimazole, prednisone and eyedrops

Patient 4: 30-year-old female	Before After	3/7 0/7	right - 14/left - 12 right - 12/left - 11	38.2; 38.4; 38.9; 38.3 36.5; 36.7; 36.7; 36.9	6 months	Methimazole and eyedrops

Patient 5: 30-year-old female	Before After	4/7 0/7	right - 19/left - 22 right - 11/left - 11	37.9; 38.6; 37.8; 38.2 36.3; 36.9; 36; 35.6	6 months	Eyedrops and thyroidectomy

Patient 6: 56-yar-old male	Before After	4/7 2/7	right - 20/left - 24 right - 15/left - 13	37.9; 37.9; 38; 38 37.1; 36.7; 36.4; 36.5	12 months	Methimazole, prednisone, eyedrops and orbital decompression surgery

Patient 7: 60-year-old male	Before After	7/7 2/7	right - 17/left - 15 right - 15/left - 12	38.8; 39.1; 37.8; 37.8 35.9; 35.7; 36.1; 36.3	12 months	Methimazole, prednisone, eyedrops and orbital decompression surgery

Patient 8: 61-year-old-male	Before After	5/7 2/7	right - 15/left - 12 right - 13/left - 12	37.9; 38.3; 37; 38.3 35.9; 36.3; 36.6; 35.1	12 months	Methimazole, prednisone and eyedrops

Patient 9: 53-year-old female	Before After	3/7 1/7	right - 15/left - 23 right - 15/left - 23	36.6; 36; 38.1; 38 35.7; 35.4; 35.7; 35.7	12 months	Methimazole and eyedrops

Patient 10: 68-year-old female	Before After	5/7 2/7	right - 22/left - 20 right - 13/left - 11	37.4; 38.5; 37.2; 38.3 36.3; 36.4; 35.4; 36.5	6 months	Methimazole, eyedrops and methylprednisolone pulse

Patient 11: 53-year-old male	Before After	5/7 5/7	right - 20/left - 24 right - 25/left - 27	38.6; 38.6; 38.3; 38.3 36.9; 36.7; 37.3; 37.9	6 months	Prednisone and eyedrops

Patient 12: 39-year-old-male	Before After	4/7 1/7	right - 21/left - 19 right - 20/left – 17	39.2; 40.1; 37.8; 38.5 35.9; 36.7; 36; 36.7	6 months	Levothyroxine, prednisone and eyedrops

## Data Availability

The data used to support the findings of this study are available from the corresponding author upon request.
